# Insulin Resistance as a Therapeutic Target in the Treatment of Alzheimer's Disease: A State-of-the-Art Review

**DOI:** 10.3389/fnins.2018.00215

**Published:** 2018-04-10

**Authors:** Christian Benedict, Claudia A. Grillo

**Affiliations:** ^1^Department of Neuroscience, Uppsala University, Uppsala, Sweden; ^2^Department of Pharmacology, Physiology and Neuroscience, University of South Carolina-School of Medicine, Columbia, SC, United States

**Keywords:** intranasal insulin, diabetes, mild cognitive impairment, amyloid beta, neurofibrillary tangles

## Abstract

Research in animals and humans has shown that type 2 diabetes and its prodromal state, insulin resistance, promote major pathological hallmarks of Alzheimer's disease (AD), such as the formation of amyloid plaques and neurofibrillary tangles (NFT). Worrisomely, dysregulated amyloid beta (Aβ) metabolism has also been shown to promote central nervous system insulin resistance; although the role of tau metabolism remains controversial. Collectively, as proposed in this review, these findings suggest the existence of a mechanistic interplay between AD pathogenesis and disrupted insulin signaling. They also provide strong support for the hypothesis that pharmacologically restoring brain insulin signaling could represent a promising strategy to curb the development and progression of AD. In this context, great hopes have been attached to the use of intranasal insulin. This drug delivery method increases cerebrospinal fluid concentrations of insulin in the absence of peripheral side effects, such as hypoglycemia. With this in mind, the present review will also summarize current knowledge on the efficacy of intranasal insulin to mitigate major pathological symptoms of AD, i.e., cognitive impairment and deregulation of Aβ and tau metabolism.

## Background

Alzheimer's disease (AD) is a devastating disease characterized by a loss or decline in memory and other intellectual functions that can lead to impairments in everyday performance. AD affects 1 in 10 people ages 65 or older, and represents 60–70% of cases of dementia (Barker et al., 2002). At the macroscopic level, brain atrophy is the key neuropathological element of AD; at the microscopic level, the hallmarks of the disease are amyloid plaques, neurofibrillary tangles (NFT), and extensive neuronal loss. The principal proteinaceous component of amyloid plaques is the amyloid beta (Aβ) peptide, a 38–43 amino acid peptide produced from the cleavage of the transmembrane amyloid precursor protein (APP) by 2 enzymes: β-secretase and γ-secretase (Golde et al., 2000; Hardy and Selkoe, 2002). The active enzymatic component of the γ-secretase complex, presenilin, cleaves APP at several sites within the membrane to produce Aβ peptides of different lengths such as Aβ38, Aβ40, and Aβ42. The Aβ aggregation process is affected by the interaction of Aβ with the Aβ binding molecules such as apolipoprotein E (apoE) in the extracellular space (Kim et al., 2009). Human apoE has three common alleles (ε2, ε3, and ε4). The ε4 allele confers a genetic risk factor for AD; conversely, ε2 allele plays a protective role (Corder et al., 1993; Strittmatter et al., 1993). Aβ clearance from the interstitial fluid (ISF) depends on molecules such as neprilysin and insulin-degrading enzyme (IDE), as well as CSF and ISF bulk flow (Qiu et al., 1998; Jiang et al., 2017). The other hallmark of AD, the NFTs, are intracellular structures composed predominantly by hyperphosphorylated tau (Grundke-Iqbal et al., 1986; Goedert et al., 1988; Wischik et al., 1988). Tau is synthesized in all neurons and is also present in glial cells. Tau is a microtubule-associated protein that binds to tubulin and stabilizes microtubules. Under physiological conditions tau phosphorylation/dephosphorylation is a dynamic process essential for tau functionality. Phosphorylation of tau induces its release from microtubules and facilitates axonal vesicle transport, when tau is dephosphorylated it binds again to tubulin (Mandelkow et al., 2004). Hyperphosphorylation of tau can be a consequence of an imbalance of tau kinase and phosphatase activity. When tau suffers a hyperphosphorylation process, the protein dissociates from microtubules and self-aggregates forming NFTs observed in cell bodies and dystrophic neurites of the patients with AD. There is strong evidence that neurofibrillary pathology contributes to neuronal dysfunction and correlates with the clinical progression of AD. It has been suggested this is likely partly through pathways downstream of Aβ. However, Aβ is not the only factor that stimulates tau deposition. Other factors such as tau levels, its sequence and its phosphorylation state also contribute to tau aggregation and toxicity (Holtzman et al., 2012). Moreover, tau-related brain damage in AD might progress independently of Aβ (Small and Duff, 2008). Recent studies of Aβ plaques and tau-related neurodegeneration showed that they progress gradually in a sequential but temporally overlapping profile (Jack and Holtzman, 2013). The presence of Aβ plaques in the brain is the first detectable biomarker, followed by CSF tau proteins; whereas the cognitive deficit is the last event in the progression of AD (Jack et al., 2013). Taking into account these parameters, it is estimated that AD pathology probably begins 10–15 years prior to cognitive decline. In other words, it takes more than one decade of protein misfolding and aggregation until substantial neurodegeneration is developed and cognitive decline shows as the main symptom of this progressing disease (Perrin et al., 2009; Jack et al., 2010). Remarkably, this gradual evolvement of the AD provides a window for early intervention.

The brain utilizes ~20% of all glucose in a process that is mainly insulin independent. However, insulin receptors are widely distributed throughout the brain, with high concentrations in the olfactory bulb, hypothalamus and hippocampus (Fernandez and Torres-Alemán, 2012). The central function of insulin receptors ranges from regulation of whole-body energy metabolism in the hypothalamus (Woods et al., 1979; Brief and Davis, 1984; Hallschmid et al., 2004; Grillo et al., 2007; Benedict et al., 2011; Thienel et al., 2017) to modulation of memory at hippocampal level (Park et al., 2000; McNay et al., 2010; Grillo et al., 2015). Similarly to AD, reductions in insulin sensitivity (i.e., insulin resistance) occur years before the patients start to experience the symptoms and are diagnosed with diabetes (Dankner et al., 2009). Insulin resistance increases AD risk by at least two-fold (Sims-Robinson et al., 2010), and this deleterious effect can be due to the disruption of the function of the brain vasculature (Biessels and Reijmer, 2014; Frosch et al., 2017), and/or direct effects on Aβ aggregation or tau phosphorylation.

In recent years, type 2 diabetes and its prodromal state, insulin resistance (a pathological condition in which cells fail to respond normally to the hormone insulin), have been identified as risk factors for developing sporadic AD. For instance, a recent meta-analysis of longitudinal population-based studies (involving 1,746,777 individuals) has shown that the risk of AD is increased by about 50% in diabetic people compared to the general population (Zhang et al., 2017). The mechanistic pathways that might link impaired insulin signaling, particularly that of the brain and AD have been subject of intensive research in recent years, and will be comprehensively reviewed herein. These findings provide strong support for the hypothesis that pharmacologically restoring brain insulin signaling could be a promising novel strategy to curb the development and progression of AD. In this context, intranasal insulin administration has emerged as a very promising therapy for AD. With this in mind, one of the objectives of the current review is to summarize clinical trials and discuss the efficacy of intranasal insulin to improve major pathological symptoms of AD, i.e., cognitive dysfunction and deregulation of Aβ and tau metabolism. Additionally, we discuss some pre-clinical and clinical studies using drugs that enhance insulin sensitivity to ameliorate AD symptoms.

## Insulin resistance, brain structure, and cognitive functions

Clinical and pre-clinical studies consistently show an association between type 2 diabetes (and its prodromal state insulin resistance) and cognitive dysfunction. Additionally, the literature shows numerous examples of cognitive improvements due to insulin treatment.

### Preclinical studies

#### AD models and insulin resistance

Insulin administration has been shown to ameliorate memory deficits and reverse diet-induced increases of Aβ levels in the brain of 3xTg-AD mice (Vandal et al., 2014). In the hippocampi of another AD mice model (APP/PS1 Tg), impairments in the insulin signaling were also reported (Bomfim et al., 2012). In addition, *in vivo* and *in vitro* experiments show that Aβ induces serine phosphorylation of insulin receptor substrate 1 (IRS-1) instead of tyrosine phosphorylation (Bomfim et al., 2012); this switch has been described as a major mechanism that triggers peripheral insulin resistance (Hirosumi et al., 2002). On the other hand, acute intrahippocampal administration of Aβ (1–42) impairs insulin signaling, decreasing phosphorylation of Akt and plasma membrane translocation of the insulin-sensitive glucose transporter 4, and these molecular effects were accompanied by deficits in spatial memory (Pearson-Leary and McNay, 2012). Although it takes from 10 to 15 years after Aβ starts to aggregate to observe cognitive impairments in AD patients, an acute effect of Aβ upon cognition cannot be ruled out, especially taking into account the disruption in insulin signaling. Whether the same mechanism applies to human brains remains to be elucidated.

#### Diabetes models and cognitive function

Experimental animal models of type 2 diabetes show impairments in hippocampal-based memory performance (Li et al., 2002; Winocur et al., 2005), deficits in hippocampal neuroplasticity including decreases in neuronal spine density and neurogenesis (Stranahan et al., 2008) and decreases in synaptic transmission (Kamal et al., 2013), whereas bolstering insulin signaling mitigates Aβ-induced synapse loss in mature cultures of hippocampal neurons (De Felice et al., 2009). Ultimately, the long-term consequences of diabetes-induced neuroplasticity deficits are reflected in cognitive impairments (Biessels and Reagan, 2015). Indeed, insulin resistance is a crucial contributor to the adverse effects on hippocampal cognitive function (de la Monte, 2012), and the literature consistently shows many examples that support the positive effects of insulin on cognitive function in rodent models. In this regard, central insulin administration improves spatial memory in a dose-dependent fashion in male rats (Haj-ali et al., 2009), whereas intrahippocampal insulin microinjections enhanced memory consolidation and retrieval (Moosavi et al., 2007). Acute delivery of insulin into the rat hippocampus also promotes spatial memory in the alternation test (McNay et al., 2010), and transiently enhances hippocampal-dependent memory in the inhibitory avoidance test (Stern et al., 2014). Nisticó et al. reported that mice with haploinsufficiency of insulin receptor β-subunit showed reduced hippocampal LTP and deficits in recognition memory (Nisticò et al., 2012). Concurrently, in a model of hippocampal-specific insulin resistance, rats showed deficits in LTP and spatial memory especially in long-term memory (Grillo et al., 2015).

### Clinical studies

#### AD and insulin resistance

Similar to preclinical studies, clinical studies show that disturbed insulin metabolism is a risk factor for cognitive dysfunction, brain atrophy, and dementia. There is evidence that insulin receptor density decreases in aging, and insulin signaling is impaired in AD (Frölich et al., 1998, 1999). In addition, post-mortem brain tissue from AD patients shows decreased insulin mRNA (Steen et al., 2005), suggesting a deficit in brain insulin signaling. Furthermore, brain tissue from AD patients without diabetes show insulin signaling impairments (Bruehl et al., 2010; De Felice and Ferreira, 2014; Yarchoan and Arnold, 2014).

Interestingly, a seminal work of Convit et al. describes memory deficits and hippocampal atrophy in individuals with impaired glucose metabolism (Convit et al., 2003). Conversion from mild cognitive impairment (MCI) to AD is higher in individuals with impaired glycemia compared to normoglycemic patients (Morris et al., 2014), suggesting that baseline glycemia and insulin resistance play key roles on cognitive decline and AD progression. Cognitive impairment is accompanied by whole-brain volume loss, although no difference was observed in hippocampal volume. In another study performed in healthy adults at risk for AD, the individuals that are strongly positive for Aβ (determined by Pittsburgh compound B tomography) show increased glucose metabolism in specific brain areas but not atrophy or cognitive loss compared to Aβ negative or Aβ indeterminate (Johnson et al., 2014). This potentially opens the opportunity to start an early intervention to prevent AD progression even in individuals that do not manifest abnormalities in peripheral glucose metabolism.

In a cross sectional study performed in cognitively healthy elderly individuals, it was shown that insulin resistance negatively correlates with verbal fluency performance and brain volume, especially in areas related to speech production (Benedict et al., 2012). However, there was no correlation when diabetic or cognitively impaired subjects were examined in an 11-year follow-up study carried out to examine a nationally representative adult population in Finland (Ekblad et al., 2017). Both studies concur that insulin resistance even in healthy individuals has a deleterious effect on verbal fluency performance. A recent cross-sectional study in late middle-aged participants at risk for AD showed that insulin resistance in normoglycemia has a positive correlation with Aβ deposition in frontal and temporal areas (Willette et al., 2015). It is important to note that these individuals are at risk for AD, whereas in previous studies in which type 2 diabetes was not associated with Aβ deposition or NFT, the brains were from patients without risk of AD (Nelson et al., 2009; Ahtiluoto et al., 2010). Furthermore, when cognitively asymptomatic middle-aged adults with a parental family history of AD were assessed, insulin resistance was associated with higher Aβ42 and long-term memory impairments (Hoscheidt et al., 2016).

When the other hallmark feature of AD, NFT, was considered, some studies suggest a link between insulin resistance and abnormal phosphorylation of tau protein (Liu et al., 2009). Insulin resistance is associated with higher P-Tau 181 and Total Tau in the CSF of asymptomatic late-middle-aged adults with risk factors for AD (APOEε4 carriers) and the association is negative for the APOEε4 non-carriers; whereas there is no effect on CSF Aβ42 levels (Starks et al., 2015). This suggests that insulin resistance may increase the susceptibility for tau pathology especially in the APOEε4 carriers.

#### Diabetic patients and AD hallmarks

Diabetes increases the odds of cognitive decline 1.2- to 1.5-fold compared to non-diabetic patients (Cukierman et al., 2005). Initial imaging studies in type 2 diabetic patients showed cortical and subcortical atrophy involving several brain regions accompanied by deficits in regional cerebral perfusion and vasoreactivity (Last et al., 2007) that ultimately may contribute to the cognitive dysfunction observed in elderly subjects with diabetes. In this regard, Crane et al. showed that higher glucose levels represent a risk factor for dementia in patients with or without diabetes. Unfortunately although hyperglycemia could result from decreases in insulin sensitivity, insulin levels were not reported (Crane et al., 2013). In a subsequent study, using glucose and hemoglobin A1c levels to characterize glucose exposure over 5 years before death, the same group did not find an association between glucose levels and NFT and dementia in people without diabetes treatment history (Crane et al., 2016). In spite of the effort to find the hallmark features of AD in the brain of type 2 diabetes patient, post-mortem studies were not able to show increased Aβ deposition or neurofibrillary tangles (Nelson et al., 2009; Ahtiluoto et al., 2010). More recent studies using Pittsburgh compound B to detect amyloid plaques mainly consisting of insoluble fibrils of Aβ—also failed to associate type 2 diabetes and Aβ aggregation (Thambisetty et al., 2013; Roberts et al., 2014). However, it must be noted that the load of insoluble Aβ does not correlate well with disease progression (Engler et al., 2006). Clinical evidence confirms that diabetes accelerates cognitive function decline, although, the mechanism still remains to be elucidated and it does not necessarily include the hallmark features of AD.

In a study performed in adults with prediabetes or early type 2 diabetes without cognitive impairment, insulin resistance was associated with reduced cerebral glucose metabolic rate (CMRglu) in frontal, parietotemporal and cingulate regions. During a memory task, individuals with diabetes showed a different pattern of CMRglu (more diffuse and extensive activation) and more difficulties in recalling items compared to healthy adults (Baker et al., 2011). This pattern is similar to that observed in prodromal or early AD as well as in non-symptomatic APOEε4 carriers; possibly these changes in CMRglu may try to compensate the disruption in the neuroarchitectural network that normally supports the cognitive task (Bookheimer et al., 2000; Sperling et al., 2010).

## Linking insulin resistance and AD: possible molecular mechanisms

### Insulin signaling pathway

**Figure 1:**
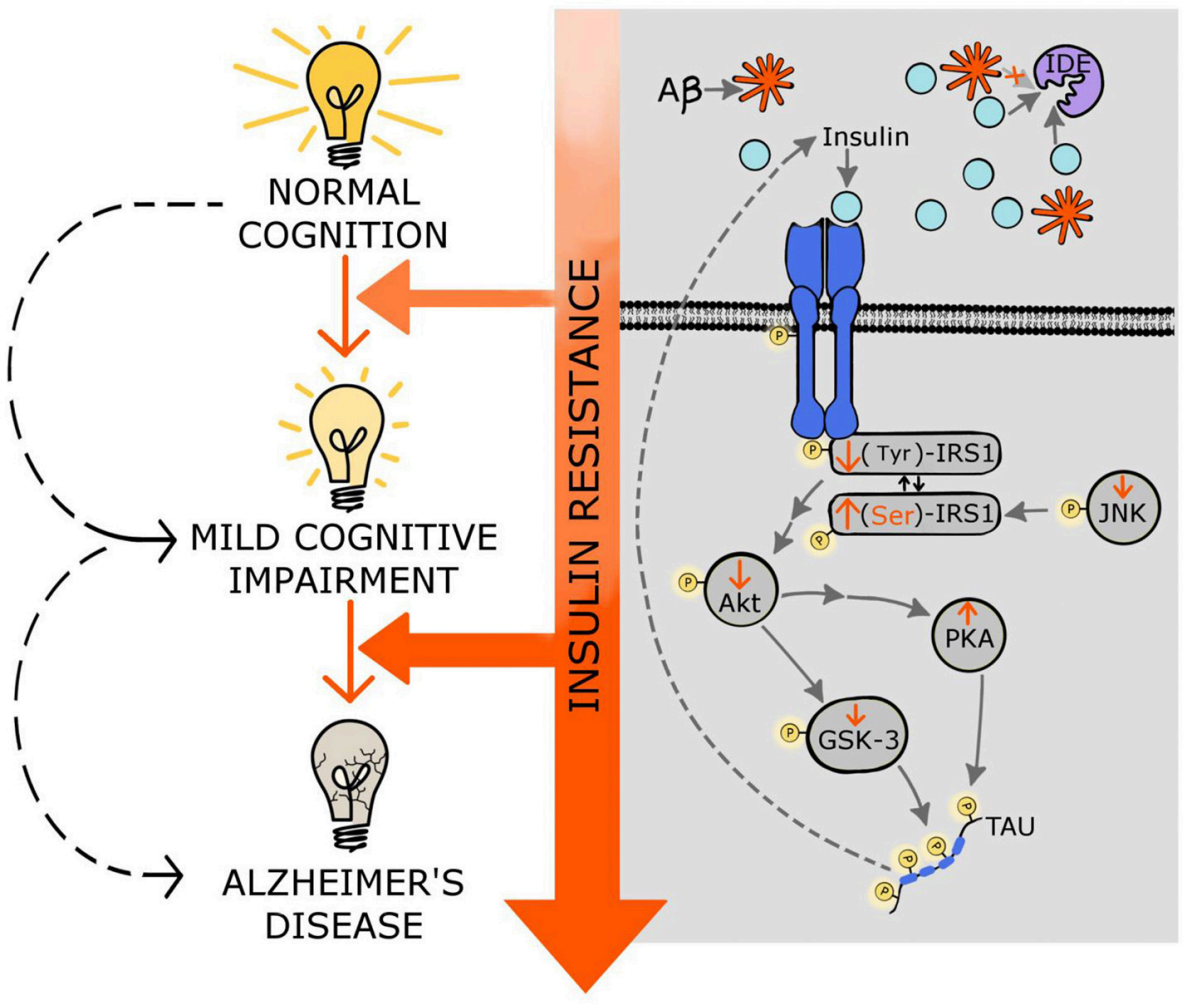
Molecular mechanisms linking insulin resistance and Alzheimer's disease. Central insulin receptors are activated similarly to peripheral insulin receptors. This activation includes the autophosphorylation of the receptor followed by phosphorylation of other components of the cascade such as IRS1, Akt, and GSK-3. Insulin resistance results in stimulation of Ser-phosphorylation of IRS1 instead of normal Tyr-phosphorylation. Additionally, insulin resistance decreases p-JNK favoring the p(Ser)-IRS1. Insulin resistance also results in decreased phosphorylation of Akt affecting several downstream components of the insulin pathway, including GSK-3; this increment of the dephosphorylated form of GSK-3 (active state) stimulates tau hyperphosphorylation and NFT formation. Moreover, decreased activity of Akt induces PKA which also contributes to tau phosphorylation. Hyperphosphorylated tau further impairs insulin signaling. Additionally, the high levels of insulin, exhibited in insulin resistance states, compete with the Aβ for the insulin degrading enzyme (IDE) that is in charge of degrading both insulin and Aβ, affecting the clearance of Aβ. Through these multiple molecular mechanisms, insulin resistance might accelerate mild cognitive impairment as well as AD development and progression. See text for details.

Although it is not clear how insulin resistance manifests in the central nervous system (CNS), many evidences suggest that different steps of the insulin signaling pathway might be altered (Biessels and Reagan, 2015). Importantly, changes in the insulin receptor expression cannot be ruled out especially in the brains of AD patients (Steen et al., 2005; Moloney et al., 2010). The first step in the insulin pathway activation, the receptor autophosphorylation, is followed by the Tyr phosphorylation of IRS1; however, in AD brains many groups reported increases in p(Ser)-IRS1, a marker of insulin resistance, instead of p(Tyr)-IRS1 (Steen et al., 2005; Moloney et al., 2010; Bomfim et al., 2012; Talbot et al., 2012; Figure 1). In addition, higher levels of p-JNK which can stimulate Ser-phosphorylation of IRS1 have been also reported in AD brains (Bomfim et al., 2012; Talbot et al., 2012; Figure 1). What leads to this switch in the insulin pathway observed in insulin resistance and AD remains to be elucidated. Recent studies from the Kapogiannis lab. show that plasma exosomes from AD patients exhibit higher pSer-IRS-1 levels and lower pTyr-IRS-1 compared to control subjects, suggesting that these biomarkers might be associated with the brain atrophy observed in AD. In fact, using neural-origin exosomes isolated by immunoprecipitation for L1 CAM, a positive correlation was observed between brain volume and pTyr-IRS-1; while the correlation was negative for pSer-IRS-1 (Mullins et al., 2017). This innovative methodology supports the hypothesis that central insulin resistance could be developed by changes in insulin signaling similarly to the changes described in the periphery and at the same time provides a potential brain-specific insulin resistance biomarker to study brain atrophy with a non-invasive and relatively simple procedure.

### Clearance and degradation of Aβ

Another possible mechanism that could explain why insulin resistance increases the risk of AD is through the clearance and degradation of Aβ. Insulin degrading enzyme (IDE) not only breaks down insulin but also degrades Aβ. In insulin resistance with high levels of insulin, IDE is saturated by insulin and it is less effective at Aβ degradation (Qiu et al., 1998; Figure 1). Clearance of Aβ is significantly decreased in rats treated with high doses of insulin (Shiiki et al., 2004). Conversely, inhibition of PI3K, a key step in the insulin pathway, suppresses APP cleavage and secretase activity, leading to decreases in Aβ production (Stöhr et al., 2013). In a mouse model of AD with neuron specific knockout of insulin receptor, Stöhr et al. (2013) observed reduction in Aβ levels and amyloid aggregation, suggesting that insulin signaling has an important effect upon Aβ deposition. In humans in a hyperinsulinemic-euglycemic clamp, insulin improved declarative memory, and increased CSF Aβ in older participants (Watson et al., 2003). In other studies using the same type of clamp, plasma and CSF Aβ was increased along with markers of inflammation (Fishel et al., 2005). These data suggest that hyperinsulinemia can regulate levels of Aβ. However, we have to take into account that these are acute effects observed after transient increases of insulin, whereas in type 2 diabetes hyperinsulinemia is chronic and therefore the long-term effect on Aβ degradation, cognitive function and AD progression could be different.

### Glymphatic clearance

An alternative mechanism by which insulin resistance exacerbates AD progression could include the clearance of the extracellular amyloid plaques. Decreases in the clearance of interstitial fluid in the hippocampus was observed in an experimental model of diabetes, and the cognitive deficits observed in the diabetic rats were inversely correlated to the retention of the contrast agent used to determine glymphatic clearance (Jiang et al., 2017). This is one of the first demonstrations that the system responsible for clearing brain extracellular solutes is affected by diabetes and might explain how insulin resistance may contribute to the initiation and progress of AD.

### Fasting insulin levels

The two extremes of fasting insulin levels (lower and upper 15th percentiles) increase the risk of dementia in a longitudinal study performed in Japanese-American elderly men (Peila et al., 2004). In both cases, lack of insulin or excess of insulin due to insulin resistance lead to the convergent development of dementia. This finding is supported by studies in rodent models in which low levels of brain insulin and impaired insulin signaling preceded Aβ aggregation in a mouse model of AD (Chua et al., 2012). In other mouse model of AD, damaging the pancreatic cells that synthesize insulin leads to increases in Aβ levels (Wang et al., 2010). However, the lack of insulin resulting from the damage of the insulin-producing cells produces hyperglycemia that can also increase Aβ aggregation (MacAuley et al., 2015; Chao et al., 2016), making it difficult to elucidate if the increases in extracellular Aβ are due to the hypoinsulinemia and/or the glucotoxicity. Interestingly, lower levels of insulin produces decreases in IDE levels with the consequent increment in Aβ deposition.

Although the majority of the studies show that central insulin resistance in AD individuals has a deleterious effect, some studies in rodents have shown that deficiency in insulin receptor signaling in the brain can have a protective effect against Aβ deposition and even can extend lifespan (Freude et al., 2009; Stöhr et al., 2013). Deletion of insulin-like growth factor-1 receptor (IGF-1R) or insulin receptor in a mouse model of AD decreases APP processing delaying Aβ aggregation. However, only IGF-1R deficiency reduces premature mortality. According to cell based experiments inhibition of the PI3-kinase suppresses APP cleavage and decreases the secretases activity. This can explain the reduction in Aβ aggregation, but the differential effect on mortality remains still unknown.

Another question that remains unresolved is the time course of potential pre-diabetes relative to AD pathology and cognitive impairment. Recent studies from MacKlin et al. (2017) showed that APP/PS1 transgenic mice exhibit glucose intolerance at 2 months of age whereas Aβ accumulation and cognitive decline are not evident until 8–9 months of age. The metabolic deficit appears earlier and persists until the AD pathology and cognitive symptoms occur, indicating that at least in this model peripheral metabolic dysregulation precedes AD pathology (MacKlin et al., 2017).

### Tau hyperphosphorylation

The hypothesis that diabetes can facilitate tau pathology through induction of hyperphosphorylation of tau is supported by different molecular mechanisms. Under physiological conditions, insulin stimulates Akt phosphorylation that subsequently leads to Ser-phosphorylation of glycogen synthase kinase 3 (GSK3) and inactivates this enzyme. The active form of GSK3 stimulates tau phosphorylation and NFT formation (Figure 1). Hyperphosphorylation of tau may induce tau missorting which can lead to synaptic dysfunction and cognitive impairments (Wang and Mandelkow, 2016). Therefore, insulin resistance reduces p-Akt and p(Ser)-GSK3, and these decreases have also been described in postmortem brain tissue from patients with AD (Steen et al., 2005; Liu et al., 2011). Conversely, other groups reported the opposite: they observed increases in p-Akt and p(Ser)-GSK3 in AD brain samples (Pei et al., 2003; Griffin et al., 2005; Yarchoan et al., 2014). Therefore, there is no consensus about the participation of the phosphorylation/dephosphorylation processes of Akt and GSK3 upon the development of tau pathologies.

The impact of insulin resistance upon tau phosphorylation and cognition remains controversial. For instance, neuron-specific insulin receptor KO mice show higher levels of phosphorylated tau due to activation of GSK3 (Schubert et al., 2004). However, these mice have no memory impairments in spite of the higher levels of p-tau. Conversely, peripheral insulin administration increased abnormal phosphorylation of tau at Ser202 in a dose-dependent fashion in the CNS (Freude et al., 2005). On the other hand, in a model of obesity-associated hyperinsulinemia without changes in glucose homeostasis, no differences were observed in tau phosphorylation, the expression of the tau-kinases and tau-phosphatases (Becker et al., 2012). Although epidemiological studies show that diabetes is a risk factor for AD, there are still discrepancies about how insulin sensitivity modulates hyperphosphorylation of tau. A recent study in mice and monkeys demonstrates that chronic hyperinsulinemia leads to hyperphosphorylation of tau (Sajan et al., 2016; Figure 1). It is important to notice that in this last study the animal models exhibit hyperglycemia whereas in the Becker's study the animals were normoglycemic. Even though insulin sensitivity plays a crucial role on AD progress; the impact of glucotoxicity upon the neurodegenerative development cannot be ruled out.

Central insulin signaling dysregulation precedes the onset of peripheral insulin resistance in two mice models of AD, Tg2576 and 3xTg-AD. However, phosphorylation of several components of the insulin signaling cascade was differentially altered in both mouse models. Whereas phosphorylation of Akt and GSK3β showed the same trend in both models, p(Ser)-IRS1 and pPI3K were increased in Tg2576 and decreased in 3xTg-AD. These differences might be due to the tau pathology developed in 3xTg-AD mice (Velazquez et al., 2017).

Recently, a new contributor to the association between insulin signaling and tau pathology has been identified. In an animal model of insulin deficiency protein kinase A (PKA), a potent tau kinase, was activated. These effects on PKA and tau phosphorylation were confirmed by *in vitro* studies (van der Harg et al., 2017; Figure 1). Interestingly, insulin administration to diabetic rats was able to reverse both effects, emphasizing the potential of insulin treatment to ameliorate taupathies including AD.

### Modulation of insulin signaling by tau

Although the effects of insulin resistance upon tau pathogenesis has been studied (for review see El Khoury et al., 2014) the effects of tau pathology upon insulin signaling has been less explored. Marcianik et al. recently proposed a new function for tau by suggesting that tau might regulate brain insulin signaling (Figure 1). This concept was based on the observation that deletion of tau impairs insulin signaling in the hippocampus. In addition the anorexigenic effect of insulin acting on the hypothalamus is disrupted in these tau knockout mice. These new findings suggest a bidirectional effect between insulin resistance and tau loss-of-function, which ultimately might impair cognitive function in AD individuals (Marciniak et al., 2017). However, it would be interesting to discern between central and peripheral insulin sensitivity since tau is also expressed in pancreatic cells and its phosphorylation/dephosphorylation play an important role in insulin trafficking and release (Maj et al., 2016).

Figure 1 depicts some of the possible molecular mechanisms that link insulin resistance with MCI and AD, and shows how the progression of insulin resistance parallels the impairments in cognitive function.

## Intranasal insulin as a treatment for alzheimer's disease

Enhancing brain insulin function has recently emerged as a possible approach to mitigate AD symptoms and pathophysiology. An effective way to centrally administer insulin is via intranasal delivery. Using this route of administration insulin travels via convective bulk flow along perivascular pathways following the olfactory and trigeminal nerves and importantly bypassing the BBB. In this way, insulin can reach the hippocampus and the cortex in 15–30 mins (Chapman et al., 2013; Lochhead et al., 2015). Importantly, intranasal insulin does not reach the peripheral circulation (Born et al., 2002), thereby avoiding peripheral hypoglycemia (for advantages and disadvantages/possible side effects of intranasal insulin administration, see Panel 1). Clinical and preclinical studies have shown beneficial effects of intranasal insulin upon Aβ aggregation, NFT and cognitive function.

**Panel 1:**
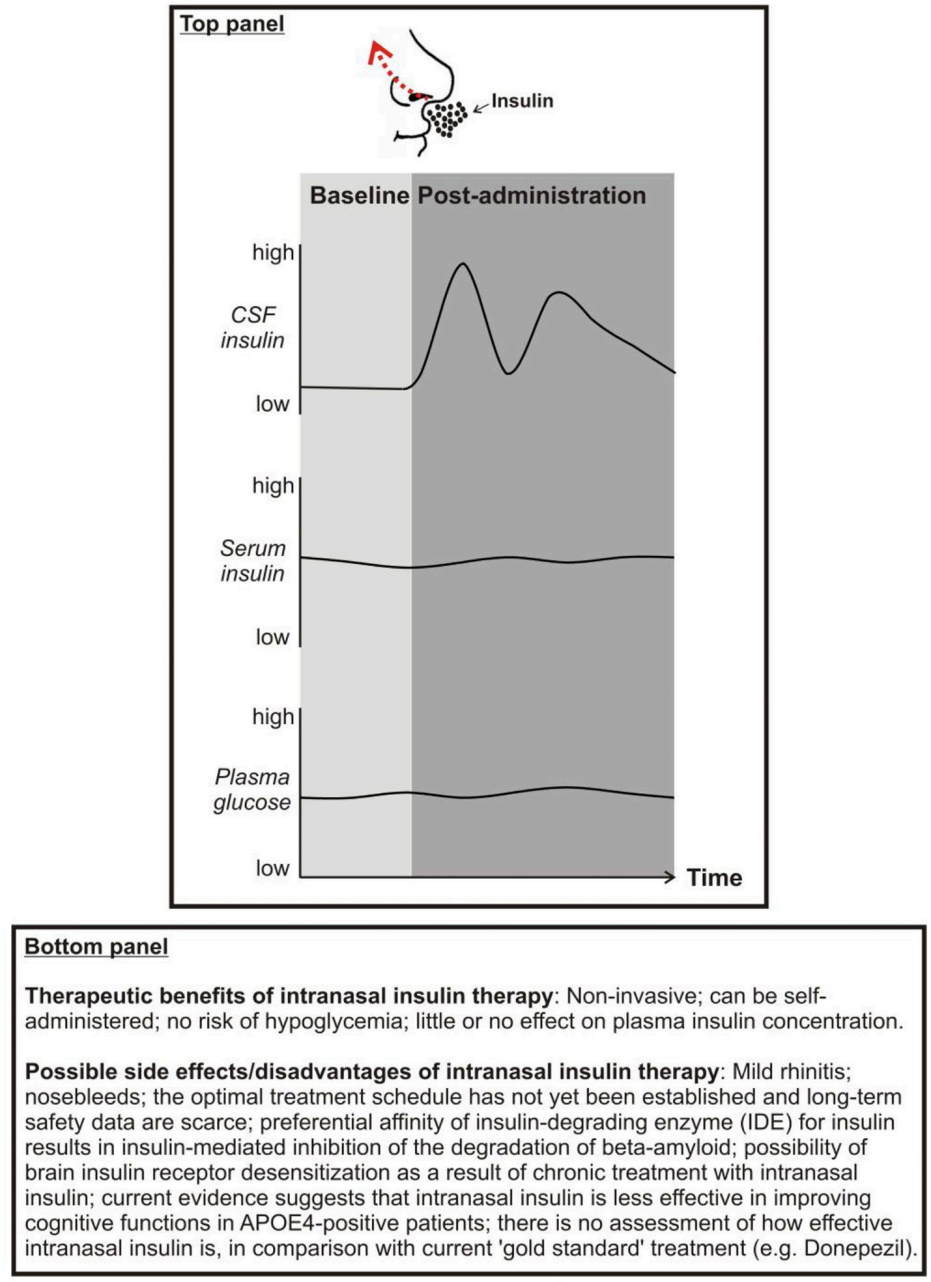
**Top**: Proposed effects of intranasal insulin on cerebrospinal fluid (CSF) insulin, serum insulin, and plasma glucose concentrations. **Bottom**: Therapeutic benefits and possible side effects of intranasal insulin.

### Intranasal insulin in diabetic and healthy individuals

In a recent study, acute intranasal insulin improved visuospatial memory in type 2 diabetic subjects as well as in the control individuals and this positive effect was due to regional vasoreactivity, especially vasodilatation in the anterior brain regions, such as insular cortex that regulates attention-related task performance (Novak et al., 2014). The same group of investigators demonstrated that intranasal insulin increases resting-state connectivity between the hippocampus and the medial frontal cortex compared to placebo and other default mode network (DMN) regions in type 2 diabetic patients. Moreover, the lower connectivity between the hippocampus and the medial frontal cortex observed in diabetic subjects was increased by intranasal insulin to a level comparable to control individuals (Zhang et al., 2015). Intranasal insulin administration was also tested in healthy individuals showing beneficial effects on cognitive function. Chronic intranasal insulin improved declarative memory (word recall) and enhanced mood (less anger and more self-confidence) in healthy male and female subjects (Benedict et al., 2004). Interestingly, these positive effects can be enhanced by using a rapid—acting insulin analog (Benedict et al., 2007). In addition to these chronic effects, transient increase of brain insulin levels improved delayed (10 min) but not immediate odor-cued recall of spatial memory in young men (Brünner et al., 2015). Interestingly, single dose intranasal insulin reduces food intake in healthy normal-weight males but not in females; conversely, hippocampus-dependent memory and working memory were improved in females, but not in males (Benedict et al., 2008). These findings could be seen as support for the hypothesis that women are more sensitive to the enhancement of hippocampus-dependent memory, whereas men are more susceptible to the anorexigenic effect of insulin. However, when obese men were long-term treated (8 weeks) with intranasal insulin although no changes were observed in body weight and adiposity, declarative memory and mood were improved similarly to normal-weight men (Hallschmid et al., 2008). It has moreover been demonstrated that intranasal insulin may normalize stress axis activity in humans by reducing cortisol levels (Benedict et al., 2004; Bohringer et al., 2008; Thienel et al., 2017). This inhibitory effect may also contribute to the positive impact on cognitive function. Finally, intranasal insulin administration has been shown to increase electroencephalogram delta power during non-rapid-eye-movement sleep in young adults (Feld et al., 2016). Sleep is a time period during which newly acquired memories are consolidated (Diekelmann and Born, 2010; Cedernaes et al., 2016) and cellular waste products accumulating in the ISF of the brain during wakefulness (such as soluble Aβ) are removed (Xie et al., 2013; Cedernaes et al., 2017). With these beneficial effects of sleep in mind, it could be speculated that intranasal insulin administration timed before sleep onset may have the strongest memory-improving and brain health-promoting therapeutic potential in humans.

#### Intranasal insulin in individuals with MCI or AD

Chronic intranasal insulin administration (4 months) in patients with MCI or mild to moderate AD improved delayed memory, preserved general cognition and functional abilities and these changes were associated with changes in Aβ42 level and tau/Aβ42 ratio in CSF. In addition, insulin impaired progression avoiding decreases in cerebral glucose metabolic rate in the parietotemporal, frontal, precuneus, and cuneus regions (Craft et al., 2012). Since no deleterious side effects were observed with this prolonged treatment, intranasal insulin emerges as an effective therapeutic approach for patients with MCI or AD. In a recent study aimed to compare regular insulin with long acting insulin (detemir) in adults with MCI or AD, the regular insulin showed improvements in memory after 2 and 4 months compared with placebo, whereas no significant effects were observed for the detemir-assigned group compared with the placebo group. Moreover, regular insulin treatment was associated with preserved volume on MRI and with reduction in the tau-P181/Aβ42 ratio (Craft et al., 2017).

#### APOE status and intranasal insulin in individuals with MCI or AD

Acute intranasal insulin improved verbal memory in ApoE ε4 negative subjects with MCI compared to ApoE ε4 positive or normal individuals (Reger et al., 2006). Interestingly and unexpectedly ApoE ε4 positive patients worsen their memory performance after insulin administration, suggesting differences in insulin metabolism due to the expression of ApoE ε4. Although both sexes were tested, gender differences were not analyzed. In a different study the same group found that repeated intranasal insulin improved verbal memory, attention and functional status compared to placebo-treated group in patients with MCI or early AD that was accompanied by increases in the short form of the beta-amyloid peptide (Reger et al., 2008b). This investigative group also found differential dose-response curves for intranasal insulin administration depending on ApoE ε4 allele: ApoE ε4 negative had a peak in verbal memory performance at 20 IU whereas ApoE ε4 positive patients showed memory decline after insulin treatment (Reger et al., 2008a). Interestingly, higher dose (60 IU) had a detrimental effect on memory in both groups (ApoE ε4 positive and negative).

A chronic study of 4 months of daily administration of intranasal insulin showed that men and women improved their cognitive function with 20 IU insulin, but just men benefited with higher dose (40 IU). When ApoE ε4 carriage was evaluated, the results showed that whereas ApoE ε4 negative men improved ApoE ε4 negative women worsened and the ApoE ε4 positive counterparts remained cognitively stable (Claxton et al., 2013). Conversely, using a long-lasting insulin analog (detemir), the results were also influenced by ApoE status; in ApoE ε4 carriers memory improvements were observed whereas non-carriers showed impairments (Claxton et al., 2015). The mechanistic basis of APOE-related treatment differences remains unknown. Collectively, these data highlight the importance of the APOE status upon the changes observed in cognition after intranasal insulin treatment. Since the treatment status can lead to beneficial or detrimental effects, it is crucial to take into account the APOE status when assessing the eligibility of the patients to participate in theses therapeutic approaches.

### Insulin sensitizer agents and AD

Since insulin has beneficial effects upon memory in individuals with or without MCI or AD, it is logical to hypothesize that drugs that increase insulin sensitivity might also have a positive effect. In this regard, members of the incretin family were considered as prime candidates to ameliorate the MCI and AD symptoms. Within the incretin family, Glucagon-like peptide-1 (GLP-1) was one of the first to be tested. GLP-1 and its receptors (GLP-1Rs) are not just expressed in the pancreas and in the vascular endothelium, they are also found in the CNS, especially in the hypothalamus, hippocampus, cerebral cortex, and olfactory bulb (Lockie, 2013). Several studies have shown the importance of GLP-1 signaling on cognitive function, and many preclinical studies have been performed to evaluate the potential protective role of GLP-1 on the brain (Calsolaro and Edison, 2015). *In vitro* Aβ oligomers impaired axonal transport and this effect was prevented by treatment with a GLP-1R agonist that is used to treat diabetes; moreover this anti-diabetes agent decreases the serine phosphorylation of IRS-1 in hippocampus improving the cognitive function in a mice model of AD (Bomfim et al., 2012). This preclinical study establishes the molecular basis to investigate the potential therapeutic effect of GLP-1 agonists to prevent or treat AD in the clinical setting.

GLP-1 analogs have a dual role: in the periphery they modulate insulin release and centrally they enhance synaptic plasticity and even are able to reverse impairments induced by Aβ oligomers (McClean et al., 2010). In addition to facilitate insulin signaling, GLP-1 analogs have neuroprotective effects *per se*. Chronic treatment with liraglutide, a long-acting GLP-1R agonist, prevented memory decline, synapse loss, synaptic plasticity impairments, decreased the Aβ aggregation, and neuroinflammation, and increased the expression of young neurons in APP/PS1 mice, suggesting that liraglutide has preventive effects at the early stage of AD (McClean et al., 2011). Interestingly, liraglutide also showed restorative effects in the later stages of the disease in 14 months-old APP/PS1 mice (McClean and Hölscher, 2014). Since liraglutide has preventive and restorative effects upon pathological hallmarks of AD, this incretin hormone has been tested in clinical trials in AD patients. Six months of liraglutide treatment did not have any effect on Aβ deposition in the temporal and occipital lobes compared to placebo-treated patients; glucose metabolism (CMRglu) decreased in placebo patients, whereas liraglutide-treated patients exhibited a trend to increase it; and cognitive function was not improved (Gejl et al., 2016). Although preclinical data were very promising in the clinical setting liraglutide failed to reverse the hallmarks of AD.

The insulin-sensitizing drug metformin, used to treat insulin resistance, was thought as a possible alternative to ameliorate the AD symptomatology. In a placebo-controlled crossover study conducted in non-diabetic patients with MCI or early AD, metformin was able to improve executive function without changes in cerebral blood flow (Koenig et al., 2017). The beneficial effects of metformin are also supported by other study that showed that 1 year of treatment improved total recall compare to baseline in overweight/obese patients with MCI (Luchsinger et al., 2016).

It is important to note that so far these insulin sensitizer agents have not been administered via intranasal route. Therefore, the efficacy of these drugs depends on the peripheral effects and the ability to cross the BBB; and these could explain the differences when compared to the intranasal insulin.

## Concluding remarks

Available evidence, as reviewed herein, suggests that central nervous system insulin resistance is frequently found in patients with AD (Freiherr et al., 2013). Worrisomely, central insulin resistance promotes major pathological hallmarks of AD that can be found in the brain long before the clinical onset of this devastating disease, such as the formation of Aβ plaques and neurofibrillary tangles (Jack and Holtzman, 2013). On the other hand, deregulated Aβ and tau metabolism has also been shown to promote central insulin resistance (Bruehl et al., 2010; De Felice and Ferreira, 2014; Yarchoan and Arnold, 2014). This suggests the existence of a mechanistic interplay between AD pathogenesis and insulin resistance. In an attempt to interrupt this vicious cycle, in recent years particular attention has been devoted to clinical trials testing effects of intranasal insulin on cognition, daily function, and AD biomarkers. This drug delivery method increases CSF concentrations of insulin in the absence of peripheral side effects such as hypoglycemia (Born et al., 2002). Collectively, results deriving from these clinical trials so far are promising in that they demonstrated beneficial effects on cognition, mood, and metabolic integrity of the brain in patients with MCI or early AD (Reger et al., 2008a,b; Craft et al., 2012, 2017; Claxton et al., 2015). However, many unanswered questions remain, such as which dose of intranasal insulin is optimal to improve cognition, preserve brain metabolism, and reduce possible side effects in AD patients? Are effects of intranasal insulin on cognition and brain health augmented when combined with insulin sensitivity-increasing interventions, such as GLP-1 infusions or exercise programs? Does the time of the day modulate central nervous system effects of intranasal insulin (e.g., morning vs. evening)? Does a chronic treatment with intranasal insulin lead to desensitization of brain insulin signaling, as seen in peripheral tissues (Kupila et al., 2003)? Notwithstanding these questions, the currently available scientific evidence provides a sufficiently strong basis for the hypothesis that counteracting insulin resistance represents a promising therapeutic target in the treatment of AD. Whether intranasal insulin represents such candidate therapy remains to be elucidated in future trials.

## Author contributions

All authors listed have made a substantial, direct, and intellectual contribution to the work, and approved it for publication.

### Conflict of interest statement

The authors declare that the research was conducted in the absence of any commercial or financial relationships that could be construed as a potential conflict of interest.
